# Effect of Differential N-linked and O-linked Mannosylation on Recognition of Fungal Antigens by Dendritic Cells

**DOI:** 10.1371/journal.pone.0001009

**Published:** 2007-10-10

**Authors:** Jennifer S. Lam, Haibin Huang, Stuart M. Levitz

**Affiliations:** 1 Department of Medicine and the Immunology Training Program, Boston University School of Medicine, Boston, Massachusetts, United States of America; 2 Department of Microbiology and the Immunology Training Program, Boston University School of Medicine, Boston, Massachusetts, United States of America; 3 Department of Medicine, University of Massachusetts Medical School, Worcester, Massachusetts, United States of America; Albert Einstein College of Medicine, United States of America

## Abstract

**Background:**

An experimental approach for improving vaccine efficacy involves targeting antigens to mannose receptors (MRs) on dendritic cells (DCs) and other professional antigen presenting cells. Previously, we demonstrated that mannosylated *Pichia pastoris*-derived recombinant proteins exhibited increased immunogenicity compared to proteins lacking mannosylation. In order to gain insight into the mechanisms responsible for this observation, the present study examined the cellular uptake of the mannosylated and deglycosylated recombinant proteins.

**Methodology/Principal Findings:**

Utilizing transfected cell lines, roles for the macrophage mannose receptor (MMR, CD206) and DC-SIGN (CD209) in the recognition of the mannosylated, but not deglycosylated, antigens were demonstrated. The uptake of mannosylated antigens into murine bone marrow-derived DCs (BMDCs) was inhibited by yeast mannans (YMs), suggesting a mannose-specific C-type lectin receptor-dependent process, while the uptake of deglycosylated antigens remained unaffected. In particular, antigens with both N-linked and extensive O-linked mannosylation showed the highest binding and uptake by BMDCs. Finally, confocal microscopy studies revealed that both mannosylated and deglycosylated *P. pastoris*-derived recombinant proteins localized in MHC class II+ compartments within BMDCs.

**Conclusions/Significance:**

Taken together with our previous results, these data suggest that increased uptake by mannose-specific C-type lectin receptors is the major mechanism responsible for the enhanced antigenicity seen with mannosylated proteins. These findings have important implications for vaccine design and contribute to our understanding of how glycosylation affects the immune response to eukaryotic pathogens.

## Introduction

Mannosylated antigens have been demonstrated to enhance MHC class I- and MHC class II-restricted antigen presentation, increase T cell proliferation, and promote T cell effector responses [1]–[3]. Such immune consequences have been attributed to the ability of particular C-type lectin receptors (CLRs) to bind and endocytose mannose-containing compounds [4]. These mannose-specific CLRs share the ability to recognize mannose residues but differ in their mannose binding affinities depending on the spatial configurations of mannose. Furthermore, different CLRs may traffic their cargo through different intracellular routes [5]–[7] but all typically result in presentation of antigen in surface MHC molecules, leading to antigen-specific T cell proliferation [5], [8], [9]. As mannose-specific CLRs are highly expressed on DCs [10] and recognize mannosylated ligands, they have become the target of vaccine strategies for eliciting antigen-specific cell-mediated immune responses.

DCs are professional antigen presenting cells that play an integral role in governing immune responses [11], [12]. Two significant methods of antigen capture by immature DCs are macropinocytosis and endocytosis that is mediated by receptors, including mannose-specific CLRs [13]. Macropinocytosis is a constitutive process in immature DCs that involves non-selective fluid phase uptake of large vesicles approximately 0.5 to 3 µM in size [13]–[15]. In comparison, MMR-mediated endocytosis selectively and efficiently captures mannosylated antigens [13]. Both mechanisms of antigen capture result in delivery to MHC class II compartments [13], [15], [16] but antigen internalized via mannose-specific CLRs are significantly more potent at augmenting in vitro T cell proliferation [1], [2], [17], [18].

Previously, we generated a panel of recombinant mannosylated proteins (summarized in Table 1) using the yeast *P. pastoris* [18]. The target antigen included in all constructs was a segment of OVA (aa 230–359) containing epitopes recognized by both CD4+ and CD8+ T cells. Two asparagines situated in the consensus motif Asn-X-Ser/Thr (where X denotes any amino acid except for proline) provided potential sites for N-linked glycans while a Ser/Thr-rich region from a cryptococcal mannoprotein designated MP98 [19] supplied sites for extensive O-linked mannosylation. For example, recombinant protein ppOVAST contained two Asn-X-Ser/Thr motifs and a Ser/Thr-rich region and therefore displayed N-linked and O-linked mannosylation. In contrast, the sequence for ppOVA only included the Asn-X-Ser/Thr motifs. Consequently, the recombinant protein ppOVA was found to exhibit mostly N-linked mannosylation and minimal O-linked glycosylation. To examine the immunostimulatory effects caused by N-linked mannosylation, we removed the sites for N-linked glycosylation using site-directed mutagenesis. This resulted in the production of recombinant proteins that lacked N-linked mannosylation but still contained O-linked glycosylation. Recombinant protein ppOVASTNQ displayed extensive O-linked mannosylation while ppOVANQ harbored minimal O-mannosylation.

**Table 1:** *P. pastoris*-derived recombinant proteins utilized in the study

Recombinant Protein	Components	Molecular Size (Unglycosylated) (Daltons)	Mannosylation Profile
ppOVA	OVA-cMyc-6XHis	17,169	N-links and minimal O-links
ppOVANQ	OVA*-cMyc-6XHis	17,197	Minimal O-links
ppOVAST	OVA-S/T rich-region-cMyc-6XHis	22,155	N-links and extensive O-links
ppOVASTNQ	OVA*-S/T rich-region-cMyc-6XHis	22,183	Extensive O-links
ppOVAST dg**	OVA-S/T rich-region-cMyc-6XHis	22,155	None

More details regarding the proteins, including schematics of glycosylation sites and representative gels, can be found in reference [18].

* Two asparagine (N) residues in the OVA portion were mutagenized to two glutamine (Q) residues, thus preventing formation of N-linked glycans.

** *P. pastoris*-produced ppOVAST was subjected to chemical deglycosylation using TFMS.

We found that all the *P. pastoris*-derived mannosylated recombinant proteins were more potent than the deglycosylated recombinant proteins in eliciting antigen-specific CD4+ and CD8+ T cell proliferation [18], [20]. Interestingly, the presence of O-linked mannosylation enhanced CD8+ T cell proliferation while N-linked mannosylation dampened these responses. However, this effect was not seen when examining CD4+ T cell responses as the presence of both N- and O-mannosylation correlated with increased CD4+ T cell proliferation. Mannose-specific CLRs appeared to play a role in inducing these elevated T cell responses as yeast mannans (YMs) inhibited CD4+ T cell proliferation. In the present study, we explored the mechanism(s) responsible for the enhanced immunogenicity of the mannosylated antigens. We hypothesized that the increased immunogenic properties of mannosylated *P. pastoris*-derived recombinant OVA proteins was due to the increased mannose-specific CLR-mediated uptake of these antigens into DCs and their subsequent trafficking into MHC class II-enriched compartments.

## Results

### Mannose-specific C-type lectin receptor binding specificities for differentially mannosylated recombinant OVA proteins

**Figure 1:**
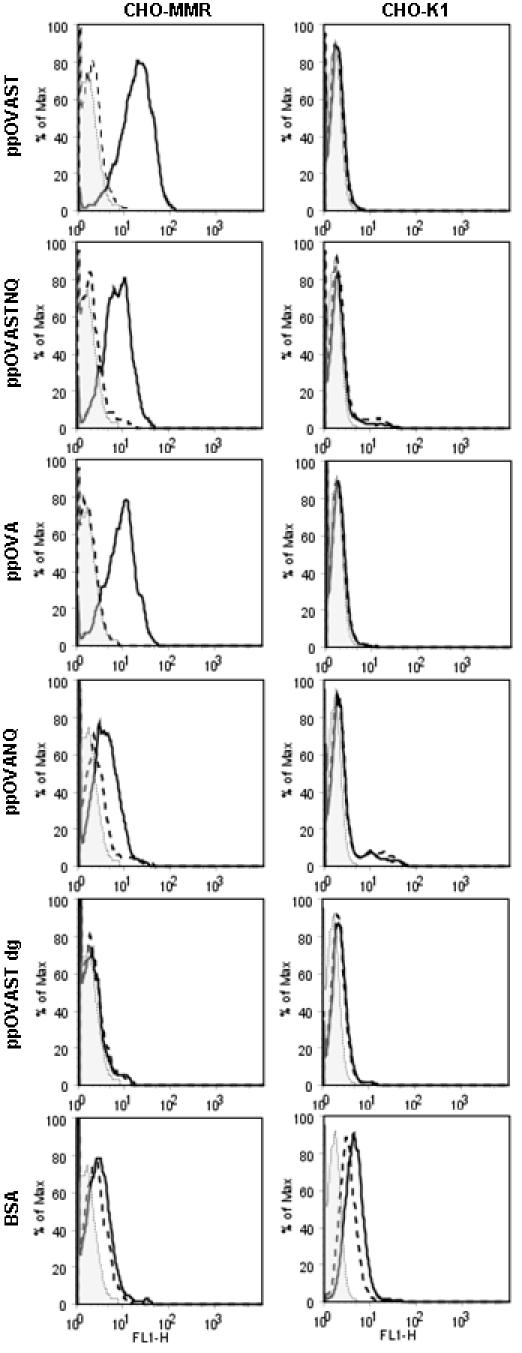
The macrophage mannose receptor recognizes *P. pastoris*-derived mannosylated antigens but not deglycosylated antigens. CHO-K1 and CHO-MMR cell lines were pulsed with OG-labeled antigens (each containing 2000 relative fluorescence units (RFU)) for 20 min in the absence or presence of YMs (1 mg/ml). Cells were harvested and analyzed for antigen binding on CHOK1 and CHO-MMR cells by flow cytometry. The CHO-MMR cells were gated on MMR+ cells by staining with PE-labeled antibodies to MMR. OG-labeled BSA served as a control for background uptake by endocytosis. Background staining of cells is indicated by the shaded histogram. Results are representative of 2 independent experiments.

**Figure 2:**
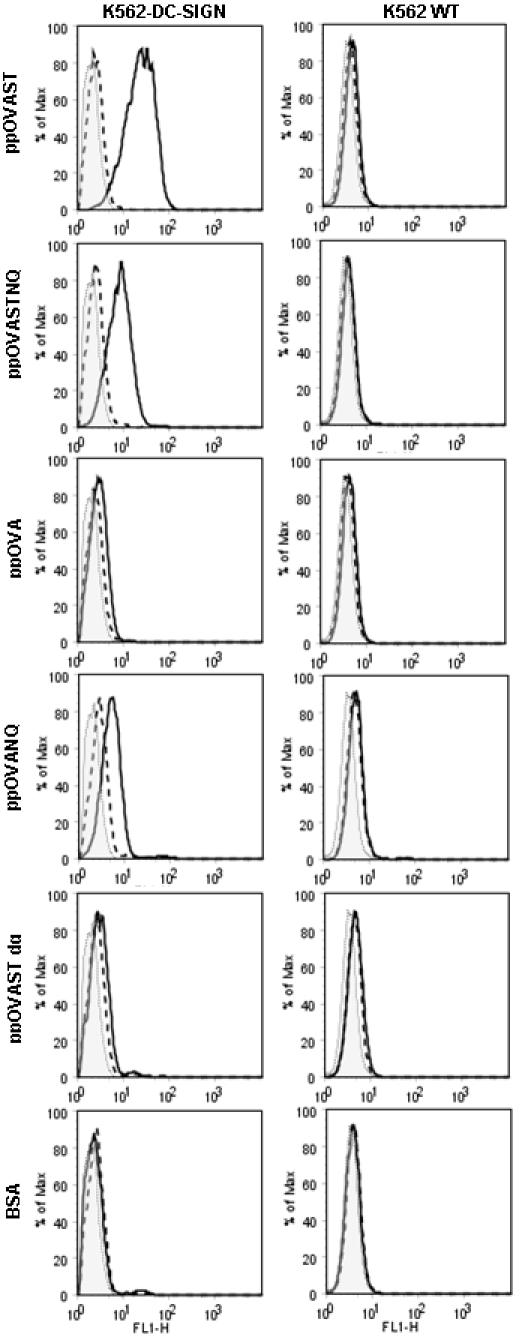
DC-SIGN recognizes *P. pastoris*-derived mannosylated antigens but not deglycosylated antigens. K562 WT and K562 DC-SIGN cell lines were studied under identical conditions as in figure 1. Results are representative of 2 independent experiments.

Our previous results suggested that the heightened antigen-specific T cell responses induced by differentially mannosylated *P. pastoris*-derived recombinant proteins were due to a mannose-specific CLR-dependent endocytic process [18]. Therefore, we sought to determine whether the mannosylated OVA antigens were capable of being specifically recognized by two major CLRs, MMR and DC-SIGN. Stably transfected cell lines expressing the MMR or DC-SIGN were incubated with Oregon Green 488 (OG)-labeled recombinant OVA antigens in the presence or absence of YMs. YMs consist of branched chains of mannose which bind mannose-specific CLRs, including the MMR [13] and DC-SIGN [5]. The concentration of YM used, 1 mg/ml, has been shown to profoundly inhibit MR [21]. Subsequent antigen binding and uptake were measured using flow cytometry. Comparative analysis of bound antigen to CHO cells expressing the MMR (CHO-MMR) and untransfected CHO-K1 controls revealed binding was strongest with ppOVAST, followed by ppOVA and ppOVASTNQ (Fig. 1). These interactions were specific as YMs were able to inhibit binding of all three aforementioned proteins (Fig. 1). In contrast, the MMR exhibited minimal to no binding to ppOVANQ, ppOVAST dg, and the endocytosis control BSA (Fig. 1). Comparison of the amount of bound antigen on K562 cells expressing DC-SIGN and untransfected K562 WT counterparts showed that DC-SIGN strongly bound ppOVAST and modestly bound ppOVASTNQ and ppOVANQ (Fig. 2). Surprisingly, ppOVA was not bound by DC-SIGN to any appreciable extent (Fig. 2). Similar to the MMR, DC-SIGN did not specifically bind to ppOVAST dg and BSA (Fig. 2). Altogether, these data suggest that optimal recognition of the recombinant proteins by MMR requires the presence of both N-mannosylation and extensive O-mannosylation while DC-SIGN relies primarily on O-mannosylation. Interestingly, cells transfected with MMR, but not DC-SIGN, had increased uptake of ppOVA.

To ascertain that the above effects were specific for CLRs with mannose-binding activity, control experiments were performed using NIH 3T3 cells stably transfected with dectin-1 [22]. Dectin-1 is a CLR which binds fungal β-glucans but not mannose [23]. Dectin-1-transfected and untransfected NIH 3T3 cells were incubated with the panel of OG-labeled antigens. Cell-associated fluorescence, as measured by flow cytometry, was nearly identical when comparing transfected and untransfected cells. Moreover, there were no significant differences amongst the individual antigen preparations, and cell-associated fluorescence was not inhibited by mannan or the dectin-1 inhibitor, laminarin (data not shown).

### Role of surface mannose-specific C-type lectin receptors in the uptake of mannosylated recombinant OVA proteins on dendritic cells

**Figure 3:**
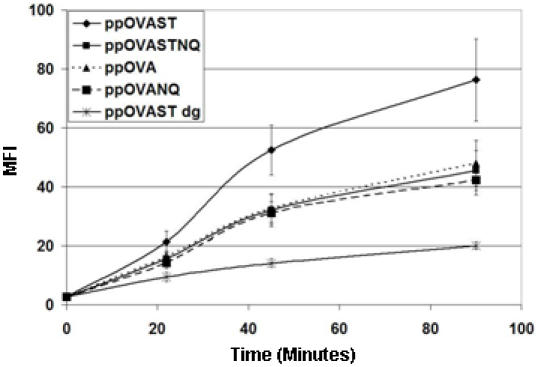
Recombinant OVA antigen uptake by DCs over time. Murine BMDCs were pulsed with OG-labeled antigens (each containing 2000 RFU) for 22.5, 45, or 90 min. Cells were washed, stained with CD11c-PE, and analyzed by flow cytometry. The MFI is represented by the geometric mean of the gated CD11c+ peak. Data represent the mean ± SEM of four independent experiments. Data were analyzed using the one-way ANOVA and Tukey's HSD post hoc test (α = 0.05). A significant increase in cell-associated fluorescence for ppOVAST was observed when compared to ppOVAST dg at all time points.

**Figure 4:**
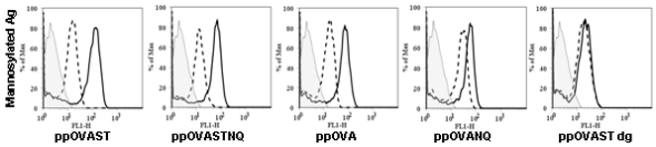
YMs inhibit surface binding and/or internalization of *P. pastoris*-derived mannosylated antigens but not deglycosylated antigens by murine BMDCs. Murine BMDCs (day 9) were pulsed with 2000 RFU of the indicated OG-labeled antigens for 90 min in the presence (dashed line histogram) or absence (heavy line histogram) of YMs (1 mg/ml). Cells were harvested, stained for CD11c-PE, and antigen uptake analyzed by flow cytometry. Histograms are gated on CD11c+ cells. Background staining of cells is indicated by the shaded histogram. Results are representative of 2 independent experiments.

We next evaluated whether DCs, which highly express both MMR and DC-SIGN [24], would differentially bind and internalize the recombinant OVA proteins. BMDCs were pulsed with OG-labeled recombinant OVA proteins for varying lengths of time and cell-associated fluorescence was analyzed by flow cytometry. As expected, binding and uptake of all proteins, as determined by an increase in fluorescence, increased as a function of time. However, BMDCs showed the greatest binding and/or internalization for the mannosylated recombinant OVA proteins when compared to the deglycosylated OVA antigens (Fig. 3). Moreover, as with the transfected cell lines, the greatest cell-associated fluorescence was observed with ppOVAST. Uptake of the macropinocytosis marker, Lucifer yellow, was similar to that of ppOVAST dg (data not shown), suggesting that ppOVAST dg is not recognized by receptor-dependent processes. To discern whether the enhanced uptake of the mannosylated OVA antigens was a mannose-specific CLR-dependent process, BMDCs were pulsed with OG-labeled recombinant OVA antigens in the presence or absence of YMs for 90 min (Fig. 4). We observed that all the mannosylated recombinant OVA antigens showed a substantial amount of cell-associated fluorescence after 90 min which was inhibited by the addition of YMs. Another antagonist of mannose-specific CLRs, α-methyl-D-mannopyranoside (100 µg/ml) [17] inhibited cell-associated fluorescence to a similar extent as did YMs, whereas the carbohydrates, N-acetyl-D-glucosamine (1 mg/ml), dextran T500 (100 µg/ml), and barley powder β-glucans (100 µg/ml) did not (data not shown). Surface antigen binding and/or uptake of pOVAST dg was not affected by any of the putative inhibitors including YMs.

**Figure 5:**
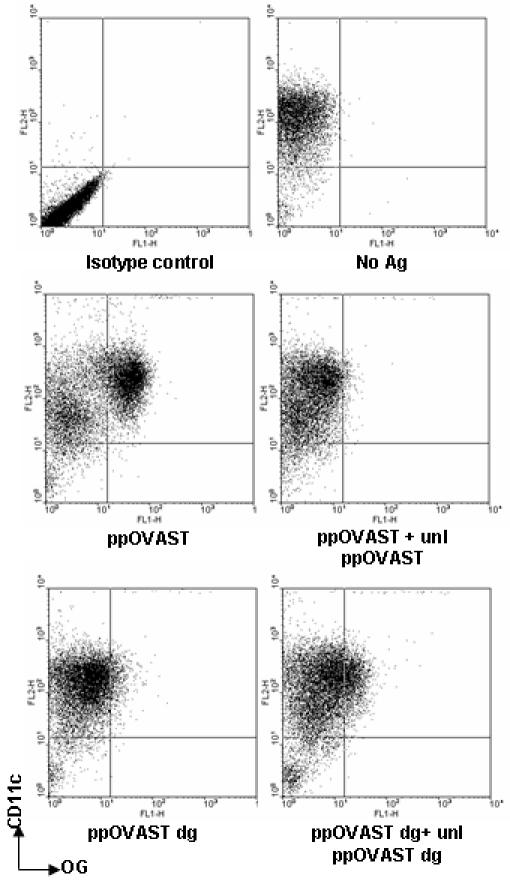
Effects of excess unlabeled recombinant OVA proteins on the uptake of fluorescent-labeled recombinant OVA proteins. Murine BMDCs were pulsed with OG-labeled antigens (each containing 2000 RFU) in the presence or absence of the same unlabeled antigen (1 mg/ml) for 45 min. Cells were washed, stained with CD11c-PE, and analyzed by flow cytometry. The plot labeled “Isotype control” represents cells treated with no antigen and stained with an isotype control antibody for CD11c-PE. Results are representative of 3 to 4 independent experiments.

The inhibitory activity of YMs on mannosylated recombinant OVA antigen uptake suggested that MRs played a predominant role in antigen internalization. To confirm that receptors were responsible for the antigen uptake and to rule out any non-specific inhibitory effects of YMs, BMDCs were pulsed with fluorescent OG-labeled antigens in the presence or absence of excess amounts of the same unlabeled recombinant OVA antigens. As would be expected of a receptor-mediated process, binding and/or uptake of fluorescently-labeled ppOVAST was inhibited by unlabeled ppOVAST to levels approaching background (Fig. 5). Unlike ppOVAST, ppOVAST dg is presumably internalized by a macropinocytic, rather than a receptor-mediated process, and therefore binding and/or internalization was not decreased by excess unlabeled ppOVAST dg. In fact, a slight enhancement in uptake was observed. Altogether, our results suggest that DCs specifically bind and internalize the mannosylated, but not the deglycosylated, recombinant OVA proteins using mannose-specific CLRs. Furthermore, DCs appear to have disparate binding affinities for the different mannosylated OVA antigens.

### Colocalization of recombinant OVA proteins and MHC class II molecules in DCs

**Figure 6:**
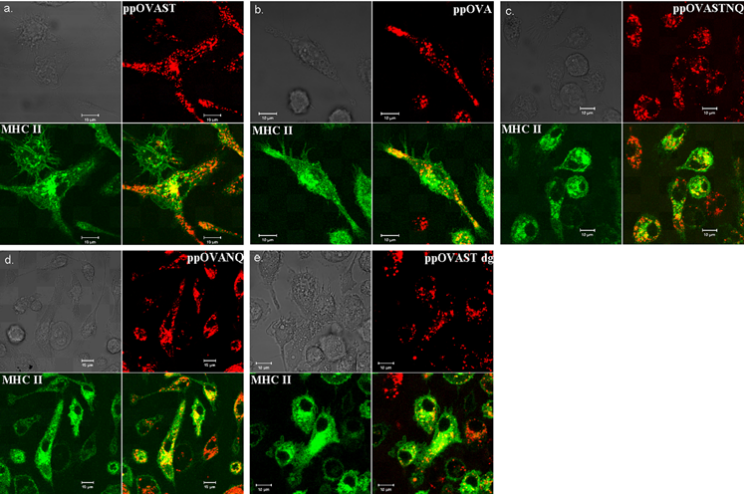
Colocalization of recombinant OVA proteins with MHC class II in murine BMDCs. MHC II-eGFP BMDCs were incubated with AF568 labeled-recombinant OVA protein (2μM) for 10 min. The live cells were then washed and kept on ice until imaging by confocal microscopy. Bar denotes 10 µM scale. Upper left: brightfield images. Upper right: red channel images demonstrating recombinant OVA proteins; Lower left: green channel images demonstrating MHC class II; Lower right: Merge images. Yellow pseudocolor indicates colocalization of recombinant OVA proteins and MHC class II. (a) ppOVAST, (b) ppOVA, (c) ppOVASTNQ, (d) ppOVANQ, (e) ppOVAST dg.

The finding that mannose-specific CLRs are involved in the uptake of mannosylated OVA antigens but not deglycosylated OVA antigens led us to consider that antigens might be sorted into distinct intracellular compartments according to their mannosylation status. In the final set of experiments, BMDCs, acquired from transgenic mice expressing MHC class II molecules (I-A^b^) tagged with enhanced green fluorescent protein (MHC II-eGFP) [25], were incubated with fluorescently labeled-recombinant OVA proteins and their subsequent intracellular fates were visualized by confocal microscopy. All the recombinant OVA proteins, including the deglycosylated versions, were found to localize in MHC II-positive compartments (Fig 6).

## Discussion

The data presented begin to address the mechanistic basis for the observation that fungal mannosylation enhances antigen immunogenicity [17], [18], [20], [26]. We focused upon the mannose-specific CLRs responsible for antigen uptake, the relative roles of N- and O-linked mannosylation, and the intracellular localization of the differentially mannosylated antigens.

Cell lines stably transfected with either MMR or DC-SIGN were used to evaluate the recognition of the differentially mannosylated OVA antigens by mannose-specific CLRs. We found that DC-SIGN and the MMR had the highest binding for ppOVAST, the antigen with both N-linked and extensive O-linked mannosylation. ppOVASTNQ, which contains extensive O-linked mannosylation, but no N-linkages, was recognized by both MMR and DC-SIGN, but with lesser binding compared to that seen with ppOVAST. In contrast, ppOVA bound to MMR but not DC-SIGN. Thus, O-mannosylation of antigen seems critical for optimal DC-SIGN binding whereas either N- or O-mannosylation of antigen appears able to act as a ligand for MMR binding. These findings were unanticipated because DC-SIGN is thought to have affinity for the type of branched high-mannose N-glycans produced by *P. pastoris*. Although DC-SIGN harbors only one carbohydrate recognition domain (CRD), DC-SIGN exists as a tetramer due to interactions of the neck domains [27]. This tetramer formation is presumed to allow binding of closely spaced glycans such as those present in N-linked high mannose oligosaccharides [27]. In contrast, the MMR is thought to exist in a single polypeptide extended formation [28] and therefore predisposes the CRDs to favor a linear arrangement of mannose residues [29], [30], such as those found in fungal O-linked glycans. As fungal O-linked oligosaccharides are generally short in length and unbranched [31], they were predicted to be better candidates for binding MMR compared with DC-SIGN. In addition, the amount of O-linked oligosaccharides may help increase the MMR affinity for the protein.

In our system, DCs were utilized as the APC for presentation of the recombinant mannosylated OVA antigens and were found to induce OVA-specific transgenic CD4+ T cell proliferation in a mannose-specific CLR-dependent manner [18]. Moreover, DCs have been identified as the major APC responsible for stimulating proliferation of cryptococcal mannoprotein-specific T cells and appear to be adept at mannosylated antigen capture via the MMR and DC-SIGN [26]. Thus, the surface expression of multiple types of mannose-specific CLRs on DCs combined with the ability of both DC-SIGN and the MMR to recognize our panel of recombinant mannosylated proteins (Fig. 2) suggest a redundancy in mannosylated antigen specificities among different CLRs. These findings help explain why no significant differences in CD4+ T cell proliferation were observed when T cells were incubated with wild-type or MMR knock-out DCs pulsed with mannosylated OVA antigens [18]. Herein, we directed our studies to specifically examine DC binding and internalization of the recombinant OVA antigens. We found that the mannosylated recombinant OVA antigens were efficiently internalized into DCs in a mannose-specific CLR-dependent manner.

The confocal microscopy data suggest that, once internalized, the mannosylated and deglycosylated recombinant OVA proteins traffic into MHC class II-containing compartments. The question then arises as to why the mannosylated antigens are so much more potent at stimulating T cell responses. Certainly, the enhanced uptake of the mannosylated antigens provides at least a partial explanation although other factors such as more efficient processing and presentation could also be contributing. Interestingly, stimulation of BMDCs with the mannosylated antigen, ppOVA, did not stimulate production of the cytokines TNFα and IL-12p70 [20]. Others have also found that while both mannose-conjugated and unmannosylated antigens colocalize with MHC class II molecules, the mannose-conjugated antigens were profoundly more potent at stimulating in vitro T cell proliferation [1], [2].

Although both are eukaryotes, a significant difference between fungi and mammals is the extensive use of protein mannosylation in the fungal kingdom. Fungal mannosylation is viewed by the mammalian immune system as a pathogen-associated molecular pattern. However, the nature of the immune response that occurs following ligation of mannose-specific CLRs by fungal mannose groups is still being defined. Our data suggesting that recognition by CLRs of N-linked and O-linked fungal mannose differs has important implications for the design of vaccines that target DC via their mannose-specific CLRs. Studies characterizing the type of immune response induced by the mannosylated recombinant OVA proteins in vivo are planned and should provide additional insights for the optimal development of mannosylated antigens as vaccines.

## Materials and Methods

### Materials and cell culture reagents

All chemical and cell culture reagents were obtained from Sigma-Aldrich (St. Louis, MO) and Invitrogen Life Technologies (Carlsbad, CA), respectively, unless otherwise stated. BMDC media consisted of RPMI 1640, 10% FBS (Tissue Culture Biologicals, Tulare, CA), 100 U/mL penicillin, 100 µg/mL streptomycin, 2 mM L-glutamine, and 55 nM β-mercaptoethanol. Cell line media were composed of RPMI 1640 containing 10% FBS, 100 U/ml penicillin, 100 µg/ml streptomycin, 2 mM L-glutamine, and 10 mM HEPES buffer.

### Recombinant mannosylated and unglycosylated OVA proteins

The generation of recombinant OVA proteins was performed as previously described [18]. Briefly, the recombinant mannosylated OVA proteins (ppOVAST, ppOVA, ppOVASTNQ, and ppOVANQ) were produced using the EasySelect Pichia Expression kit (Invitrogen Life Technologies, Carlsbad, CA). Mannosylated recombinant OVA proteins were purified over a Ni-NTA (Qiagen, Valencia, CA) column using fast performance liquid chromatography (AKTA model; Amersham Pharmacia Biotech, Piscataway, NJ). Proteins were lyophilized, resuspended in PBS, and filter-sterilized. Chemical deglycosylation of ppOVAST was performed using the GlycoProfile IV chemical deglycosylation kit (Sigma-Aldrich, St. Louis, MO) as previously described.

#### Primary Cells

Bone marrow-derived DCs (BMDCs) were generated following the protocol of Lutz et al [32] as in our previous studies [18]. Briefly, bone marrow was obtained from tibiae and femurs of 8–14 week old male C57BL/6 mice. Cells were seeded at 2×10^6^ per 100 mm bacteriological Falcon Petri dish in 10 mLs of BMDC media supplemented with 10% GM-CSF-containing supernatant from the J558L cell line. Cell cultures were fed on day 3, 6, and 8 and used on day 9 or 10.

#### Cell lines expressing CTL receptors

CHO cells stably transfected with the MMR (CHO-MMR) and K562 cells stably transfected with DC-SIGN (K562 DC-SIGN) (a gift from Dr. A. Corbi, Madrid, Spain) were maintained in cell line media with 0.5 mg/ml Geneticin as in our previous studies [17], [26]. NIH 3T3-Dectin-1 [22] and the parent cell lines, NIH 3T3, CHO-K1 and K562, were maintained in cell line media.

### Mice

C57BL/6 male mice were purchased from The Jackson Laboratory, Bar Harbor, ME. MHC II-eGFP mice were a gift of J. Vyas and H. Ploegh, Cambridge, MA. These transgenic mice express enhanced green fluorescent protein-tagged MHC class II molecules [25]. Animals were housed under pathogen-free conditions at the Boston University Laboratory Animal Science Center. All animal experiments were conducted with approval by the Boston University Institutional Animal Use and Care Committee.

### Fluorescent labeling of recombinant proteins

Proteins were dialyzed against a sterile solution of 0.1 M sodium bicarbonate buffer (pH 8.3) and adjusted to a final concentration of 1 mg/ml. Fluorescent dyes (Oregon Green 488 (OG) or Alexa Fluor 568 (AF568)) (Invitrogen Life Technologies, Carlsbad, CA) were dissolved in anhydrous DMSO and added to the recombinant proteins at a final concentration of 15 µg/ml. Samples were incubated for 3 to 4 hours at room temperature or overnight at 4°C protected from light. Unreacted excess fluorescent dye was removed by dialysis using Ultrafree Centrifugal Filter Devices with a molecular weight cut-off membrane of 10K (Millipore, Billerica, MA). A Tecan GENios fluorescent plate reader equipped with Magellan software (Tecan Trading AG, Switzerland) was used to read and analyze the relative fluorescence values obtained from each sample. Protein concentration was assessed using the BCA assay (Pierce). The efficiency of labeling was not significantly affected by mannosylation (data not shown). Samples were stored in the dark at 4°C.

### Binding/Uptake Assays Using Fluorescent-Labeled Proteins

Recombinant proteins were pre-incubated with polymyxin B (20 µg/ml) to neutralize potential endotoxin contamination. Binding/uptake of recombinant proteins or BSA by BMDCs was not altered by polymyxin B (data not shown). BMDCs (day 9 or 10) or cell lines (CHO-K1, CHO-MMR, K562, K562-DC-SIGN, NIH 3T3, NIH 3T3-Dectin-1) were then incubated with fluorescent-labeled proteins at 37°C for the indicated amounts of time. Cells were washed 3 times with DPBS containing 2% FBS. Samples were then stained with the desired antibody and analyzed using flow cytometry.

### Confocal Microscopy

BMDCs (day 10) were resuspended at 5×10^5^ cells/ml in BMDC media supplemented with 10% GM-CSF containing supernatant from the J558L cell line and plated at 6×10^4^ cells in glass-bottom confocal dishes (MatTek, Ashland, MA). DCs were allowed to adhere overnight. Fluorescent-labeled antigens mixed with polymyxin B (at a final concentration of 20 µg/ml) were added to the DCs for 10 minutes. Cells were gently washed 3 times with ice cold BMDC media and kept on ice until confocal imaging. DCs were then visualized using a Zeiss inverted LSM 510 confocal laser scanning microscope equipped with an argon and two helium-neon lasers. Confocal images were analyzed using Zeiss LSM510 software.
